# Preserving Mediterranean Donkeys: A Study on Milk Production and Nutritional Benefits

**DOI:** 10.3390/ani14243713

**Published:** 2024-12-23

**Authors:** Mohamed Aroua, Antonella Fatica, Samia Ben Said, Mokhtar Mahouachi, Elisabetta Salimei

**Affiliations:** 1 Laboratoire d’Appui à la Durabilité des Systèmes de Production Agricoles du Nord-Ouest, Ecole Supérieure d’Agriculture du Kef, Université de Jendouba, Complexe Universitaire Boulifa, Le Kef 7119, Tunisia; sabensaid@gmail.com (S.B.S.); taymallahmah@gmail.com (M.M.); 2 Dipartimento Agricoltura, Ambiente e Alimenti, Università degli Studi del Molise, Via de Sanctis snc, 86100 Campobasso, Italy; salimei@unimol.it

**Keywords:** donkey, milk, lactation, Wood model

## Abstract

The aim of the study was to model lactation curves and assess the physicochemical properties, amino acid, and fatty acid profiles of milk from two Mediterranean donkey populations, Masri (n = 14) and North African (n = 14), using the Wood model. Over a lactation period of 205 ± 12.5 days, North African donkeys produced more milk (188.66 ± 7.19 kg) than Masri donkeys (163.42 ± 7.21 kg, *p* < 0.05). Peak milk yields occurred on day 57 for North African donkeys (1.212 kg/day) and day 59 for Masri donkeys (0.991 kg/day), with similar persistency indices of 7.19 and 7.21, respectively. North African donkey milk had significantly higher protein (1.45 ± 0.03 g/100 g) and β-lactoglobulin (4.75 ± 0.06 mg/mL) contents, while Masri donkey milk contained more fat (1.16 ± 0.05 g/100 g). Amino acid analysis revealed higher glutamate (0.27 ± 0.12 g/100 g) in North African donkey milk, while Masri donkey milk had more aspartate (0.16 ± 0.04 g/100 g). North African donkey milk had higher palmitic acid (20.1 ± 0.07 g/100 g), while Masri donkey milk had more oleic acid (21.4 ± 0.42 g/100 g). Lactation curve fitting yielded R^2^ values of 93.8% for Masri donkeys and 95.7% for North African donkeys. These findings suggest that both populations are well-suited for milk production, particularly for human consumption or food applications. North African donkeys exhibited superior yields and nutrient profiles suitable for functional food applications.

## Simple Summary

This study investigated the milk production and quality of two breeds of Mediterranean donkeys, Masri and North African. Donkey milk is known for its potential health benefits, making it valuable for people with allergies and for infant nutrition. The research aimed to compare the amount of milk produced by each breed and analyze the nutritional content, such as proteins, fats, and essential nutrients. Results showed that North African donkeys produced more milk than Masri donkeys. Additionally, North African donkey milk contained more protein and certain beneficial nutrients, while Masri milk had a higher fat content. The study also examined the levels of important amino acids and fatty acids, which are essential for health. North African milk had similar saturated fats, while Masri milk had more unsaturated fats, which are considered more beneficial for cardiovascular health. These findings suggest that although both breeds can produce milk suitable for human consumption, North African donkeys may be more suitable for producing milk with a higher nutritional value. This research could enhance the use of donkey milk as a healthy food option, contributing to the enhancement of rural economies by promoting sustainable farming practices.

## 1. Introduction

The number of donkeys in the world is around 50 million heads; these are unequally distributed, as 50% of the population is located in Ethiopia, Sudan, Pakistan, Tchad, and Mexico [1]. Agricultural mechanization is reported as the main cause of the significant reduction in donkeys, leading to the extinction of some breeds, especially in European and South American countries [2].

Besides their traditional use for stool, transport, and trade, donkeys are today included in pet therapy plans and have recently been exploited for the production of milk and its derivatives, for food and cosmetic industries, and even for ethnopharmacology [3]. Donkey milk presents many virtues and similarities with human milk [3,4,5] and has been used for infant nutrition, especially for children who develop allergies to proteins in cows’ milk [6,7]. Asinine milk has a crude protein content averaging 2%, consistent with human milk [8]. The nitrogen fraction is composed of casein (50%) and whey protein (38%), which are lower and higher, respectively, than those reported in cow’s milk [5,9,10]. The remaining 12% of the nitrogen fraction consists of non-protein nitrogen (NPN), a standard milk component. This fraction includes urea, free amino acids, and small peptides. It is important to note that the non-protein nitrogen fraction in donkey milk is generally higher than in cow’s milk. The composition of whey proteins highlights the richness of donkey milk in lysozyme and immunoglobulin, which have been related to the antibacterial, antimicrobial, antitumor, and antioxidant properties of this milk [3,11,12,13,14]. On the other hand, donkey milk is also rich in lactose, which makes it more palatable, as well as in vitamins and minerals [15,16].

In Tunisia, donkeys have been used for a long time for mule production [9]. Tunisian asinine population, according to different Authors, ranges from 123,067 [17] to 241,000 [1] heads. The Tunisian donkey population is mainly composed of three ecotypes: North African, Masri, and Arabian. The North African ecotype, which is the most widespread, represents 70% of the population and is characterized by a bay color and an average withers height ranging from 115 to 120 cm. The Masri breed, which accounts for 20% of the population, is a dwarf breed with a withers height of less than 110 cm, and it has a white color with the presence of dorsal and ventral stripes. The Arabian ecotype is the least common, making up only 10% of the donkey population. It is known for its large size and withers height of over 125 cm, and is characterized by a black color and the presence of stripes.

The majority of studies evaluating donkey milk production and quality were undertaken in European countries using very different breeds managed under very distinct conditions [10,18,19]. Some of these studies have even been carried out using intensive system management [3] since donkey milk is an emerging dairy sector in Europe. This new dairy donkey farming system was first developed in Italy and then extended to other areas around the world. However, studies on donkey milk produced in Southern Mediterranean areas, characterized by peculiar pedo-climatic conditions, are limited. Moreover, unlike cows, studies on donkey lactation curve modeling are rare in the literature [4,19,20]. Parametric models are largely used due to their limited mathematical complexity and ability to fit large curves [21]. Many equations are used in lactation curve modeling and, generally, they are classified into five-parameter models (polynomial equations) or three-parameter models, including the incomplete gamma function of Wood [22] and Wilkmink [23]. For overall curves of lactation, especially with frequently collecting data, the Wood (WD) model is suggested for the dairy equine [19].

This research aims to contribute to the advancement of knowledge regarding donkey milk by evaluating the kinetics of lactation curves of the two Tunisian donkey populations, namely Masri and North African, using the Wood model. The quality of milk of the two autochthonous populations, which have already undergone phenotypic and genetic characterization [9], is also investigated. 

## 2. Materials and Methods

### 2.1. Informed Consent

The Review Board of the Laboratoire d’Appui à la Durabilité des Systèmes de Production Agricoles du Nord-Ouest at the Ecole Supérieure d’Agriculture du Kef, Université de Jendouba, approved the research protocol including farming conditions imposed by the Tunisian law (The Livestock Law No. 2005-95 of 18 October 2005).

### 2.2. Milk Sampling

Donkey milk was sampled from 28 multiparous lactating asses belonging to two Tunisian donkey populations, Masri (n = 14) and North African (n = 14). Animals that were on average 8 (±0.7) years old and 192 (±8.6) kg in body weight were raised according to a semi-intensive breeding system in a commercial dairy donkey farm located in El Kef, Tunisia (36°10′56″ N, 8°42′53″ E).

The experiment took place after foaling, from March to December. Lactating donkeys were kept on maquis shrubland and fed concentrates as a dietary complement, as described by Aroua et al. [24]. Asses were hand milked twice a day, at 10 a.m. and 3 p.m., emptying the mammary glands as completely as possible. After the afternoon milking (3 p.m.), the foals were kept with their dams until 5 a.m., i.e., 5 h before the following morning’s milking, which is the same time between the two milking intervals. It is important to note that all efforts were made to ensure that the mammary glands were completely emptied before removing the foals.

For each jenny individual milk yields from both milkings were recorded once a week starting from the second week until the end of lactation (205 ± 12.5 days). Once a week, milk was also individually sampled after thorough homogenization and stored in a sterile tube (30 mL) containing 0.3% 2-Bromo-2-nitro-1,3-propanediol. Tubes were stored under refrigerated conditions (4 °C) and transported to the laboratory for chemical analyses. All analyses were performed in triplicate within 24 h of sample collection.

### 2.3. Chemical and Physicochemical Analyses

The pH values, total solid, fat, crude protein, non-protein nitrogen, casein, whey protein, lactose, ash contents, amino acids, and fatty acid composition of the milk samples were investigated.

The pH values were measured using a digital pH meter (model-HI 98107 pHep HANNA Instruments, Carrollton, TX, USA). Total solids were determined by oven drying at 103 °C to constant weight, and total ash content was gravimetrically determined after incineration at 530 °C, according to the Association of Official Analytical Chemists [25]. The polarimetry method, based on measuring the specific rotation of polarized light using chiral molecules, was used to measure the lactose content [26].

The International Dairy Federation standard methodology [27] was followed to evaluate the contents of crude protein (CP), true protein (TP), caseins, whey proteins, non-casein-nitrogen (NCN), and non-protein nitrogen (NPN). The total nitrogen content was multiplied by a factor of 6.38 to obtain CP. The TP content was measured by processing the milk samples with 12% trichloroacetic acid. The conversion factors of 3.60 and 6.25 were used to convert the nitrogen (percent) to the contents of NPN and NCN, respectively. Protein fractions (nitrogen) were computed as follows:

TP=CP−NPN

$$Casein\left(N\%\right)=Total\;protein\left(N\%\right)-NCN\;(N\%)$$

Whey Protein=NCN−NPN

#### 2.3.1. Lysozyme and Whey Protein Contents

Whey proteins were extracted according to Rafiq et al. [28]. The lysozyme and whey proteins α-lactalbumin and β-lactoglobulin contents were quantified by HPLC using a reversed-phase column (RP-HPLC HPLC chain Ultimate 3000, Dionex, Sunnyvale, CA, USA) on column C84.6 × 150, 5 µm (Zorbax 300SD-C8, Agilent, Santa Clara, CA, USA ), according to the literature [29].

#### 2.3.2. Amino Acid Profiles

Amino acid profiles were analyzed according to the procedure described by [28]. Milk samples were mixed and hydrolyzed using HCl 6 M for 22 h at 110 °C. The hydrolyzate was centrifuged and the filtered supernatants (0.22 μm) were used for amino acid analysis. The amino acid profiles were determined using HPLC on a resin column exchange (20 × 0.46 cm i.d.) using a Biochrom 30 series AA analyzer (Biochrom Ltd., Cambridge Science, Park, UK). The elution was carried out in a pH gradient, according to the literature [30].

#### 2.3.3. Fatty Acid Profiles

The fat content of milk was extracted using the suggested technique of chloroform-methanol extraction [31]. According to the IUPAC technique, methyl esters were produced by direct transesterification [32]. Capillary gas chromatography was used to analyze the fatty acid profile of milk samples. The analyses were carried out using an AGILENT 6890 N gas chromatograph (AGILENT 6890 N, Santa Clara, CA, USA) equipped with a flame-ionization detector and a WCOT fused-silica capillary column (CP-Sil88 100 m × 0.25 mm × 0.20 µm film thickness) under the same analytical conditions as previous studies [33]. The separation was carried out at the following temperatures: 60 °C for 2 min; 150 °C for 12 min at an 8 °C/min rate; 175 °C for 20 min at a 2 °C/min rate; 225 °C for 10 min at a 5 °C/min rate; 240 °C for 10 min at a 10 °C/min rate. The carrier gas was hydrogen (flow rate: 1 mL/min). The detector temperature was 260 °C and the injector temperature was 255 °C (splitting ratio, 50). A comparison analysis using standard reference was used to identify fatty acid peaks. The fatty acid content was given as g/100 g of fatty acids detected.

### 2.4. Statistical Analysis

The measurements were presented as average values per sampling day with their corresponding standard deviations obtained from three separate analyses. To compare the physicochemical milk quality and milk yield between the two donkey populations, a one-way analysis of variance was performed using the Xlstat Addinsoft (2016.02.27444) [34]. Statistical significance was defined at *p* < 0.05. The average milk yield, fat, protein, lactose, and total solids percentages for both populations of donkeys were evaluated to test the fit of the WD model [22]:

$$Y\left(t\right)=a\times t^{b}\times e^{-ct}$$

where Y = milk yield (kg/d); t = lactation day (d); and a, b, and c = parameters that define the scale and shape of the lactation curve.

The main lactation curve traits, including peak day (b/c), peak yield (a × (b/c)^b^ × e^−b^), and persistency (s = −(b + 1) × ln(c)), were calculated for the WD model using parameter combinations based on [35]. The total milk yield at 205 days was obtained for Masri, North African, and total investigated populations by summing the predicted daily yields. The performance of model fitting was compared using the R^2^ value. The presence of serial autocorrelation in the residuals was evaluated using the Durbin–Watson (DW) statistic.

The total milk production of individual lactations was calculated using the official Fleischmann method, as described by D’Alessandro and Martemucci [35], with the following formula:

$$Y=y_{1}{\times t}_{1}+\sum \frac{\left[(y_{i}+y_{i+1}\right)}{2}\times (t_{i+1}-t_{1})]$$

where Y represents the total milk production, y_1_ is the milk yield on the first test day, y_i_ is the milk yield on the i-th test day, t_1_ is the time (in days) from foaling to the first test day, t_i_ is the time (in days) from foaling to the i-th test day, and i ranges from 1 to k − 1, representing the total number of test days.

This method accurately calculates the total milk yield over the lactation period by integrating the yields across multiple test days.

## 3. Results

### 3.1. Kinetics of the Lactation Curves of Masri and North African Donkey Populations

**Figure 1:**
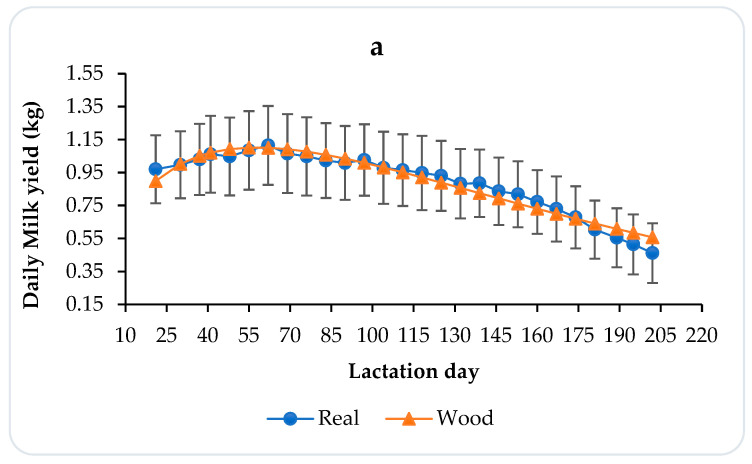

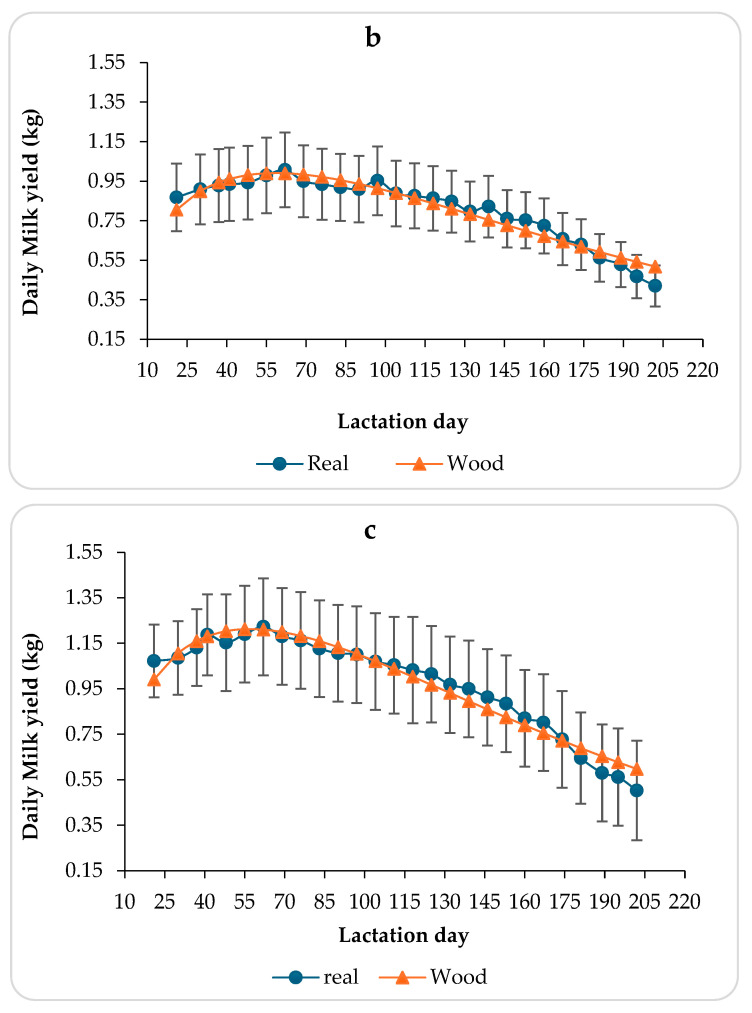
Real and WD estimated lactation curves of milk-harvested data for total donkey (**a**), Masri (**b**), and North African (**c**) populations.

Lactation curves and estimated WD model parameters of total donkey, Masri, and North African populations are represented in Figure 1 and Table 1, respectively.

This pattern involves a gradual increase in milk production after foaling, followed by a gradual decrease until lactation ends.

The initial production is higher in North African than in Masri donkeys, as reported in Table 1. The peak production day occurred at the same time in both genotypes, at about 60 days from parturition, with a peak yield higher in the North African breed (Table 1 and Figure 1). The two populations showed a good persistency index (7.19 and 7.21), with the estimated total milk production higher in the North African population (188.66 kg) than in the Masri one (163.42 kg), which is an indicator of the jennies’ ability to keep up milk production over time and showed interesting results for all populations. This indicates that the jennies retained a high level of consistency of milk production throughout the lactation period.

The DW statistics indicated the existence of a strong positive autocorrelation between residuals for the WD function. The coefficients of determination (R^2^) showed interesting values of over 90% for all donkey population curves for the WD function.

### 3.2. Characteristics of Masri and North African Donkey Milk

The harvested yield, chemical, and physicochemical characteristics of Masri and North African donkey milk are summarized in Table 2.

As reported in Table 2, milk from North African donkeys exhibits a higher protein content and lower fat content (*p* < 0.05) compared to milk from Masri donkeys. Additionally, North African donkeys have a significantly greater total milk yield (*p* < 0.05).

North African donkey milk has a similar NPN/total protein ratio compared to Masri donkey milk (*p* > 0.05) but it contains higher concentrations of β-lactoglobulin and lysozyme (*p* < 0.05). Masri donkey milk has higher levels of α-lactalbumin (*p* < 0.05). The other investigated milk parameters do not show significant differences between the two asinine populations (Table 2).

The amino acid composition of donkey milk is reported in Table 3. Except for glutamate, aspartate, and the sum of total AA (*p* < 0.05), the two Tunisian populations show comparable amounts of the other predominant amino acids: leucine, valine, serine, lysine, and proline (Table 3).

The fatty acid composition of Tunisian donkey milk (Table 4) reveals that the two most prominent fatty acids are oleic acid (C18:1, n-9) and palmitic acid (C16:0). Compared to the Masri breed, North African donkey milk contains more butyric (C4:0, *p* < 0.05), palmitic (C16:0, *p* < 0.01), margaric (C17:0, *p* < 0.01), stearic (C18:0, *p* < 0.01), and linoleic (C18:2, n-6, *p* < 0.05) acids, but lower levels of caproic (C6:0, *p* < 0.01), caprylic (C8:0, *p* < 0.01), capric (C10:0, *p* < 0.05), lauric (C12:0, *p* < 0.05), myristoleic (C14:1, *p* < 0.05), palmitoleic (C16:1, *p* < 0.05), heptadecenoic (C17:1, *p* < 0.05), oleic (C18:1; n-9, *p* < 0.05), and linolenic (C18:3, n-3, *p* < 0.05) acids. As reported in Table 4, the total unsaturated fatty acids are lower in milk from the North African population (*p* < 0.05) than in milk from the Masri population; however, the ratios UFA/SFA and n6:n3 are not significantly different.

### 3.3. Lactation Curve Modeling of Chemical Components

**Figure 2:**
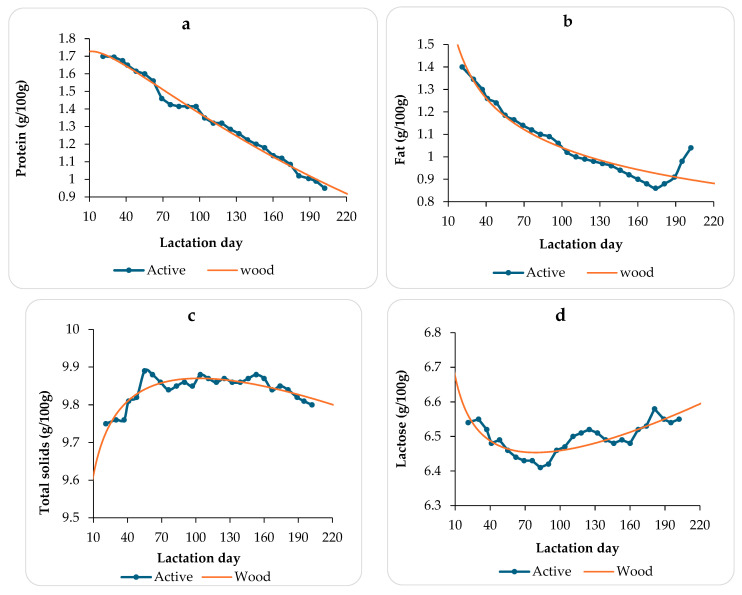
Active (Real) and WD estimated protein (**a**), fat (**b**), total solids (**c**), and lactose (**d**) curves.

Milk protein, fat, total solids, and lactose curves estimated by the Wood model on the total donkey population are represented in Figure 2 and Table 5.

The research findings illustrated in Figure 2 reveal intriguing patterns in the nutrient composition of donkey milk throughout the lactation period. Specifically, the protein and fat contents exhibited parallel trends, peaking at the beginning of lactation and then steadily declining as lactation progressed. However, the fat content showed an increase in concentration towards the end of lactation, which is to be further investigated as possibly related to the weaning process. Conversely, the total solids and lactose contents were relatively constant during lactation (Figure 2).

As shown in Table 5, the Wood model is suitable for estimating both fat and protein contents, as evidenced by high R^2^ values of 92.6% and 98.9%, respectively.

However, it is worth noting that the DW statistics indicated the presence of strong positive autocorrelation between residuals for the WD functions related to fat and protein content. This autocorrelation suggests that there might be some underlying patterns or dependencies in the residuals that the models did not fully account for. This could be indicative of certain factors or variations not adequately captured by the model.

## 4. Discussion

The North African and Masri donkey populations have shown a notable aptitude for milk production; however, values of daily milk production from the North African and Masri populations are lower than values from Martina Franca [35] and Ragusano [36] breeds. The fluctuation in milk production can be attributed to various factors, including breed, management of foals and dams, milking system and management, stage of lactation, foaling season, and parity [5,36]. Understanding and accounting for these factors is essential for optimizing milk production in donkey populations, ensuring welfare and efficient management practices for foals and dams.

The lactation curves of the North African and Masri donkey populations exhibit a consistent pattern, characterized by a gradual increase in milk production after foaling, followed by a gradual decrease until the end of lactation, as observed in the Ragusano breed [36,37].

The Wood model, considered to be the most suitable model for estimating milk yield and lactation curves [35,37], shows for the North African and Masri ecotypes the same R^2^ and logic lactation parameters. Similar results were reported for the Ragusano breed [20] and Marina Franca jennies [4]. The peak day of milk production was reached over 50 days after foaling in both populations, suggesting a consistent trend in the physiological response of jennies within a similar time frame. Kaskous and Pfaffl [37] reported that most female donkeys reach their maximum milk yield between 64 and 73 days of lactation. It is worth noting that previous studies have also shown that the highest milk production usually occurs between 30 and 60 days of lactation [10]. These findings may differ from the Littoral Dinaric breed (<30 days) because of genetic effects [38]. The persistency index in our study was 7.2 for all Tunisian donkeys. This parameter exceeded the value of 6.3 in the Ragusano breed [20] but was comparable to the value of 7.0 observed in the Martina Franca breed [19]. The persistence of lactation yield is an important determinant of total milk yield. According to De Palo et al. [19], a high persistency index in equids indicates a good aptitude for milk production.

Regarding modeling the nutrient contents, the WD model showed promising results for estimating protein and fat contents, achieving the highest R^2^ values. This finding aligns with results shown by previous studies [20,37], indicating the reliability and effectiveness of the WD model in predicting protein and fat contents in this context. Throughout the lactation period, the protein and fat contents in donkey milk exhibit a similar pattern, peaking at the beginning of lactation and gradually decreasing until reaching their minimum levels by the end of lactation. In contrast, the dry matter and lactose contents maintain consistent levels throughout lactation. These variations in nutrient content are likely influenced by dietary factors and environmental conditions, in line with findings from other studies [38].

The pH of donkey milk from Masri and North African populations is consistent with published values [9,39,40], confirming that neither breed nor lactation stage affects milk pH. The milk’s total solid values for the two donkey populations are comparable to those reported for other breeds [41,42] but higher than those observed (8.62 g/100 g) in the circum-Mediterranean breed [43].

Masri and North African donkey milk had comparable ash contents, consistent with data reported in the literature for other donkey breeds [3,5,8,10]. Salimei and Fantuz [8] indicated that equine milk is less rich in ash compared to cow, sheep, and goat milk. The ash content reported for donkey milk should be considered positively from a nutritional standpoint. Moreover, donkey milk is reported to be generally rich in Fe and Zn, as is human milk; however, dietary mineral supplementation did not increase its content [41].

The average lactose concentration of the two Tunisian donkey populations is comparable to that reported previously [38,42,43]. The high lactose content contributes to the milk’s pleasant taste and promotes the intestinal absorption of calcium, which is required for the baby’s growth. Lactose is a valuable source of galactose and glucose involved in the development of the nervous system [40,43].

Milk protein contents from Tunisian donkey populations observed in this work were consistent with the range of 1.3 to 1.9 g/100 g reported by [44]. The protein content in donkey milk is much lower than in cow milk [8]. In the nitrogen fraction, NPN represented 11% and 13% of total nitrogen content in North African and Masri donkey milk, respectively. These results fell within the range of values (10–16%) reported by [8], who also observed that the main compound in the NPN fraction is urea (40%).

The casein fraction in milk from both asinine populations is close to that in human milk, lower than that in cow milk (60 to 80% of the total nitrogen fraction), and in line with values reported in the literature for donkey milk [8,42,44]. The low casein content and casein/whey protein ratio for donkey milk have an essential impact on the sensitization potential of the milk in children with cow’s milk protein allergy [45].

Whey proteins, abundant in donkey milk protein fraction, are reported to be beneficial in the skin aging reformation process [46], as well as in reducing irritation [29,47]. Two isoforms of α-lactalbumin from donkey milk exist, each with a distinct isoelectric point [48]. Alpha-lactalbumin has recently been shown to have antiviral, anticancer, and antistress effects [49,50]. For instance, research on human breast milk demonstrates that α-lactalbumin can induce tumor-selective apoptosis by combining with oleic acid to produce Human Alpha-lactalbumin Made Lethal to Tumor Cells (HAMLET) [51]. This chemical aspect could be considered for the potential anti-proliferative activity of donkey milk [52]. Additionally, it has been demonstrated that α-lactalbumin has anti-inflammatory properties that are mediated through the inhibition of COX-2 and phospholipase A2 [53].

Milk from North African asses contains more β-lactoglobulin than that from Masri donkeys and cow milk (3.75 mg/mL) [44]. The predominant whey protein in cow milk, β-lactoglobulin, which is missing in human milk, is considered one of the main protein allergens in children [54]. Notwithstanding the observed absolute value of β-lactoglobulin, it is worth noting that in donkey milk, it accounts for about 40% of the whey proteins, which is lower than that of cow milk and comparable to mare milk [55]. Furthermore, donkey milk β-lactoglobulin is a monomer, whereas cow milk β-lactoglobulin is a dimer. The protein β-lactoglobulin belongs to the lipocalin family and has a high affinity for a diverse range of chemicals, leading to a variety of hypotheses about its function. This protein has been shown to play an important role in hydrophobic ligand transport and assimilation, enzyme activity, and the newborn acquisition of protective immunity [56]. These features might be connected to donkey milk’s hypoallergenic properties [54].

The donkey milk lysozyme content may eliminate or decrease intestinal illnesses in newborns [57,58]. Furthermore, donkey milk’s high lysozyme concentration does not affect probiotic strain growth or acidification activity, making it an excellent substrate for the creation of probiotic fermented milk drinks [59].

The donkey milk protein fraction had an amino acid profile in line with values reported in the literature [5]. Additionally, the cysteine content is confirmed to be lower than in human breast milk [8].

The fat content of donkey milk from North African and Masri populations was consistent with recent research on Tunisian donkeys [9,24], but it was found to be higher than data reported for the circum-Mediterranean breed by Charfi et al. [43] (0.72 g/100 g) and Papademas et al. [44] (0.5–0.7 g/100 g). The disparity observed in donkey milk might be attributed to various factors, including the lactation stage [5] and the milking method and strategy [42]. The low-fat content of donkey milk leads to gross energy content consistent with data reported in the literature but lower than values reported for both human and cow milk [8]. The low-energy content warrants a careful evaluation of donkey milk’s inclusion in babies’ diets [60].

The milk SFA compositions of the two Tunisian donkey populations are consistent with data reported by D’Alessandro et al. [35] for the Martina Franca breed but are lower than those reported by Salimei and Fantuz [42] for the Martina Franca and Ragusana breeds. The differences between donkey milk can be caused by different experimental protocols, breeding systems, pasture characteristics, lactation stages, parities, and health statuses [61]. The high monounsaturated fats (MUFAs) content in donkey milk has been demonstrated to have beneficial effects on human health [62,63], including the fibrinolytic mobility of surging plasma, which helps to regulate vascular endothelial physiology, as mentioned by Pérez-Jiménez [64]. In contrast, SFA intake has been linked to an increase in cardiovascular risks [65].

The lipid fraction in Tunisian donkey milk was characterized by a high amount of linoleic and linolenic acids (Table 5). These findings are similar to those reported in the literature [42,44] and confirm the similarities with the fat content of human milk in terms of high essential fatty acid levels, low saturated fatty acid contents, high levels of polyunsaturated fatty acids with a more balanced n-6:n-3 ratio, and a high level of unsaturated/saturated fatty acid ratio, as influenced by the dietary fat composition [42,44]. Donkey milk can be used as a functional food in human nutrition for infants and the elderly compared to cow’s milk and offers several health benefits related to inflammatory diseases such as dermatitis, rheumatoid arthritis, and cancer [47].

## 5. Conclusions

The North African and Masri donkey populations show a good aptitude as dairy breeds whose milk is characterized by an equivalent nutritional quality compared to other donkey breeds. The Wood model was confirmed to be suitable for predicting donkey milk yield and protein and fat contents. Donkeys may help boost the inner Mediterranean’s micro-economies, as donkey milk’s importance has recently grown due to its multidisciplinary applications in various sectors, i.e., dairy, medicine, cosmetics, and infant food when its physicochemical composition is properly integrated into the diet. Donkey milk is an intriguing product because it contains a variety of protective factors that may benefit consumers.

Even though donkey milk has so many benefits, its consumption is limited due to both a lack of information and adverse myths. The health-promoting properties of donkey milk necessitate further investigation with an interdisciplinary approach. More research and proper communication of the results could help dispel the misconceptions and promote its use, contributing to the revitalization of the ecosystem services of marginal areas.

## Figures and Tables

**Table 1:** Lactation curve parameters and characteristics estimated by the Wood (WD) model.

Population	a ^1^	b	c	Total Yield (kg)	Persistency	Peak Yield (kg)	Peak Day	DW	R^2^
Masri	0.190	0.537	0.009	163.42	7.21	0.991	59	0.555	93.8
North African	0.226	0.553	0.010	188.66	7.19	1.212	57	0.523	95.7
Total donkey	0.208	0.545	0.009	175.62	7.20	1.10	58	0.446	95.1

a ^1^ = factor representing the initial average milk yield per sampling day; b and c = parameters that define the scale and shape of the lactation curve; DW = Durbin–Watson statistics.

**Table 2:** Average daily chemical and physicochemical characteristics of milk from North African and Masri donkey populations (means ± SD) over the course of the entire lactation period.

	North African	Masri	Significance
Milk harvested, kg/day	0.97 ± 0.20	0.89 ± 0.15	*
Ash, g/100 g	0.49 ± 0.06	0.45 ± 0.05	NS
Total protein, g/100 g	1.45 ± 0.03	1.32 ± 0.05	*
Fat, g/100 g	0.90 ± 0.02	1.16 ± 0.05	*
Lactose, g/100 g	6.54 ± 0.13	6.52 ± 0.09	NS
Casein, g/100 g	0.69 ± 0.02	0.63 ± 0.05	NS
NPN, g/100 g	0.18 ± 0.04	0.20 ± 0.02	NS
Whey protein, g/100 g	0.58 ± 0.04	0.49 ± 0.05	NS
α-lactalbumin, mg/mL	1.62 ± 0.06	1.78 ± 0.08	*
β-lactoglobulin, mg/mL	4.75 ± 0.06	3.60 ± 0.03	*
Lysozyme, mg/mL	1.50 ± 0.03	1.20 ± 0.01	*
Casein/whey protein ratio	1.19 ± 0.22	1.28 ± 0.15	NS
Casein/total protein ratio	0.44 ± 0.04	0.47 ± 0.02	NS
Whey protein/total protein ratio	0.40 ± 0.02	0.37 ± 0.01	NS
NPN/total protein ratio	0.11 ± 0.01	0.13 ± 0.01	NS
pH	7.05 ± 0.02	7.02 ± 0.02	NS

NS = not significant; * = *p* < 0.05.

**Table 3:** The amino acid profiles (g/100 g) of North African and Masri donkey milk (means ± SD).

Amino Acid	North African	Masri	Significance
Glu	0.27 ± 0.12	0.23 ± 0.09	*
Leu	0.15 ± 0.06	0.08 ± 0.04	NS
Asp	0.14 ± 0.06	0.16 ± 0.04	*
Val	0.13 ± 0.04	0.09 ± 0.02	NS
Ser	0.12 ± 0.02	0.11 ± 0.03	NS
Lys	0.11 ± 0.06	0.10 ± 0.08	NS
Pro	0.10 ± 0.05	0.13 ± 0.02	NS
Ile	0.07 ± 0.01	0.08 ± 0.02	NS
Tyr	0.07 ± 0.01	0.04 ± 0.02	NS
Ala	0.07 ± 0.02	0.06 ± 0.01	NS
Arg	0.06 ± 0.01	0.06 ± 0.01	NS
Phe	0.05 ± 0.01	0.04 ± 0.01	NS
Thr	0.04 ± 0.001	0.05 ± 0.001	NS
His	0.03 ± 0.012	0.03 ± 0.014	NS
Met	0.02 ± 0.014	0.03 ± 0.011	NS
Gly	0.01 ± 0.002	0.01 ± 0.004	NS
Cys	0.002 ± 0.001	0.004 ± 0.001	NS
Total	1.45 ± 0.03	1.32 ± 0.05	*

NS = not significant; * = *p* < 0.05.

**Table 4:** The fatty acid composition (g/100 g of total FA) of donkey milk from North African and Masri populations (means ± SD).

Fatty Acid	North African	Masri	Significance
C4:0	0.56 ± 0.12	0.36 ± 0.08	*
C6:0	0.46 ± 0.08	0.90 ± 0.09	**
C8:0	5.70 ± 0.12	7.20 ± 0.18	**
C10:0	13.1 ± 0.22	14.5 ± 0.19	*
C12:0	7.62 ± 0.14	8.20 ± 0.16	*
C14:0	5.23 ± 0.21	5.30 ± 0.14	NS
C15:0	0.42 ± 0.05	0.31 ± 0.06	NS
C16:0	20.1 ± 0.07	16.5 ± 0.02	**
C17:0	0.41 ± 0.04	0.20 ± 0.06	**
C18:0	2.10 ± 0.20	1.10 ± 0.05	**
C20:0	0.11 ± 0.04	0.09 ± 0.01	NS
C22:0	0.03 ± 0.00	0.03 ± 0.00	NS
C12:1	0.14 ± 0.04	0.12 ± 0.01	NS
C14:1	0.61 ± 0.05	0.72 ± 0.06	*
C16:1	2.13 ± 0.06	2.40 ± 0.06	*
C17:1	0.31 ± 0.03	0.45 ± 0.08	*
C18:1	20.2 ± 0.50	21.4 ± 0.42	*
C18:2 n6	13.2 ± 0.52	11.9 ± 0.32	*
C18:3 n3	6.78 ± 0.09	7.32 ± 0.22	*
C20:2 n6	0.23 ± 0.04	0.20 ± 0.05	NS
C20:5 n3	0.21 ± 0.02	0.25 ± 0.08	NS
C22:6 n3	0.31 ± 0.08	0.27 ± 0.05	NS
SFA	55.6 ± 0.45	55.0 ± 0.32	NS
UFA	44.1 ± 0.26	45.0 ± 0.37	*
UFA/SFA ratio	0.79 ± 0.16	0.69 ± 0.12	NS
n6:n3 ratio	1.84 ± 0.24	1.54 ± 0.21	NS

NS = not significant; * = *p* < 0.05; ** = *p* < 0.01.

**Table 5:** Total solids, protein, fat, and lactose curve parameters estimated with the WD model.

Content	a ^1^	b	c	DW	R^2^
Lactose	7.097	−0.028	0	0.852	68.2
Total solids	9.225	0.019	0	1.273	75.9
Protein	1.619	0.044	0.004	0.867	98.9
Fat	2.718	−0.208	0	0.399	92.6

a ^1^ = factor representing the initial average content per sampling day; b and c = parameters that define the scale and shape of the lactation curve; DW = Durbin-Watson statistics.

## Data Availability

The original contributions presented in the study are included in the article, further inquiries can be directed at the corresponding author.
